# GLP-1 receptor agonists and delayed gastric emptying: implications for invasive cardiac interventions and surgery

**DOI:** 10.1097/XCE.0000000000000321

**Published:** 2024-12-04

**Authors:** Aditi Shankar, Aditi Sharma, Ariel Vinas, Robert J. Chilton

**Affiliations:** a Division of Cardiology, Department of Medicine, University of Texas Health San Antonio; b U.S. Department of Veteran Affairs, Audie L Murphy Veteran’s Association Hospital, San Antonio, Texas, USA

**Keywords:** anesthesia, gastroparesis, glucagon-like peptide-1 receptor delayed gastric emptying

## Abstract

Glucagon-like peptide-1 (GLP-1) is a hormone involved in glucose homeostasis and satiety regulation. The review highlights the importance of understanding the interplay between GLP-1 and gastric motility. This paper explores the intricate connection between GLP-1 and delayed gastric emptying, specifically gastroparesis, and its implications in the context of pulmonary aspiration during anesthesia along with the potential effects of GLP-1 medications on absorption of other medications. The findings noted in this paper serve as a catalyst for continued exploration into the intricate dynamics of GLP-1 and its implications in the context of perioperative care, aiming to enhance patient safety and optimize anesthesia practices. The inquiry suggests that an in-depth examination of this relationship is crucial for refining perioperative management strategies. It underscores the need for further research to elucidate the mechanisms involved and to establish guidelines that address the potential risks associated with GLP-1 modulation, particularly in patients undergoing anesthesia for various cardiac surgeries and procedures. Specifically in the context of cardiac interventions understanding the potential for delayed absorption of critical cardiac medications due to the influence of GLP-1 on gastric emptying is particularly important as drug absorption can play a crucial role for ensuring successful outcomes.

## Introduction

Glucagon-like peptide-1 (GLP-1) is a hormone that plays a pivotal role in maintaining glucose homeostasis by enhancing insulin secretion and inhibiting glucagon release after meals. In addition to its many effects on glucose metabolism, it also influences various physiologic processes, including the regulation of appetite, gastrointestinal motility, and cardiovascular benefits [1].

In the context of anesthesia and aspiration risks, concern revolves around the possibility of gastric contents entering the respiratory system during the anesthetic process. Aspirations during anesthesia can lead to severe complications such as pneumonia, with factors like a full stomach, compromised protective airway reflexes, and reduced consciousness, thus theoretically heightening the risk in patients taking GLP-1 medications compared with the general population. Additionally, within this context, many cardiac medications such as antiplatelets can come with a significant risk of bleeding which can also affect outcomes in surgeries.

## Delayed gastric emptying and gastroparesis

GLP-1 is a hormone that is released in response to food intake and plays a role in delaying gastric emptying, aiding in regulating blood glucose levels by moderating nutrient absorption from the stomach to the small intestine. GLP-1 also contributes to reduced appetite by inducing a sense of satiety and inhibits gastric acid secretion, beneficial in conditions of excess gastric acidity.

These effects on gastric motility have implications for the treatment of diabetes, where GLP-1 receptor agonists are now increasingly employed to manage blood sugar levels. However, individual responses to these therapies can vary, and healthcare providers need to consider these factors when prescribing and monitoring medications.

**Fig. 1:**
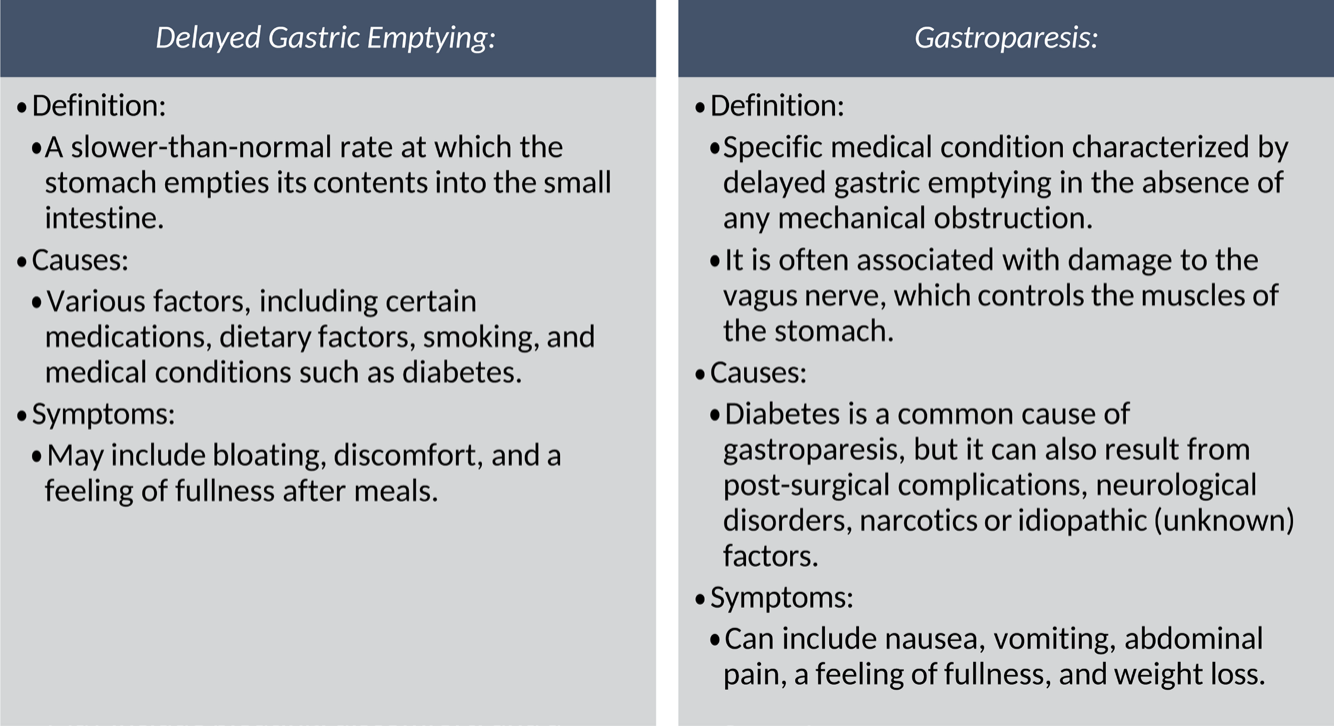
Contrasting definitions, causes, and symptoms of delayed gastric emptying and gastroparesis.

Delayed gastric emptying, where the stomach empties slower than normal, can be caused by a variety of factors leading to symptoms such as bloating and fullness. Gastroparesis, a form of delayed gastric emptying, often involves vagus nerve damage. The cause is often attributed to diabetes. Commonly experienced symptoms include nausea, vomiting, and abdominal pain. In short, delayed gastric emptying is a broader term that can be a symptom or result from various factors, while gastroparesis specifically refers to a condition where there is delayed gastric emptying due to impaired stomach muscle function, often involving damage to the vagus nerve. These conditions are further detailed in Fig. 1.

Gastroparesis has a diverse etiology, encompassing over 90 identified causes. However, a substantial percentage of patients (between 30 and 50%) have the condition with an unknown cause, termed ‘idiopathic’ gastroparesis. Common contributors to gastroparesis include systemic and metabolic disorders, with prevalent factors being diabetes (25%), medications (22%), postsurgical reasons (7%), and additional factors such as Parkinson’s disease, connective tissue disorders, and specific infections such as enteroviruses [2].

Diagnosis and management of these conditions typically involve a combination of clinical evaluation, imaging studies, and sometimes specialized tests such as gastric emptying studies. Treatment approaches may include dietary modifications, medications to stimulate gastric motility, and in severe cases, more invasive interventions such as surgery.

## Mechanisms in which glucagon-like peptide-1 may influence gastric emptying and gastroparesis.

### Delayed gastric emptying

Studies suggest that the GLP-1 receptor may have a role in regulating gastric emptying. GLP-1 receptor agonists, designed to mimic the effects of GLP-1, may slow down gastric emptying. This effect could be beneficial in conditions where delayed gastric emptying, such as in gastroparesis, is a concern.

### Neurological effects

GLP-1 receptors are present not only in the pancreas but also in the central and peripheral nervous systems, including the enteric nervous system of the gastrointestinal tract. The activation of these receptors may influence nerve signals that control gastric motility [1].

### Appetite regulation

GLP-1 is involved in the regulation of appetite and satiety. Medications that increase GLP-1 activity have been shown to influence gastric emptying times [3].

### Gastric emptying times: glucagon-like peptide-1

In a double-blind study conducted in 2013, the impact of daily dosing of liraglutide on 5-h gastric emptying scintigraphy was assessed. Liraglutide was administered over a 5-week period, revealing a significant increase in gastric retention at 1-h. However, this difference normalized between the liraglutide and placebo groups by the 5-h mark [4]. Additionally, Van Can *et al*. [4] reported that the area under the curve (AUC) for gastric emptying from 0 to 300 min was comparable between liraglutide 1.8 and 3.0 mg, as well as between liraglutide and the placebo.

Nevertheless, reductions in gastric emptying were observed at the 1-h mark, with liraglutide 3.0 mg showing a 23% decrease and liraglutide 1.8 mg exhibiting a 13% decrease compared with the placebo [2].

In another study, a double-blind, parallel-group design was employed to investigate the impact of semaglutide on gastric emptying in 72 adults diagnosed with obesity. Participants were randomly assigned to receive either once-weekly subcutaneous semaglutide (with dose escalation up to 2.4 mg) or a placebo over a 20-week period. Gastric emptying was evaluated by measuring paracetamol absorption following a standardized breakfast. The administration of 2.4 mg of semaglutide demonstrated a notable 8% increase in paracetamol AUC 0–5 h compared with the placebo [5].

In a placebo-controlled trial involving 24 healthy participants, Delgado-Aros *et al*. [6] investigated the impact of GLP-1 administered intravenously on stomach volume using 99mTc-single photon emission computed tomography imaging. The study also monitored how GLP-1 affected the gastric processing of a liquid nutrient meal (Ensure) by using scintigraphy, assessed the maximum volume of Ensure that participants could tolerate, and recorded any pos-tmeal symptoms experienced 30 min after reaching this maximum volume. Additionally, the study looked at how GLP-1’s effects might be related to the vagal cholinergic system by measuring the human pancreatic polypeptide response after consuming the Ensure meal.

**Fig. 2:**
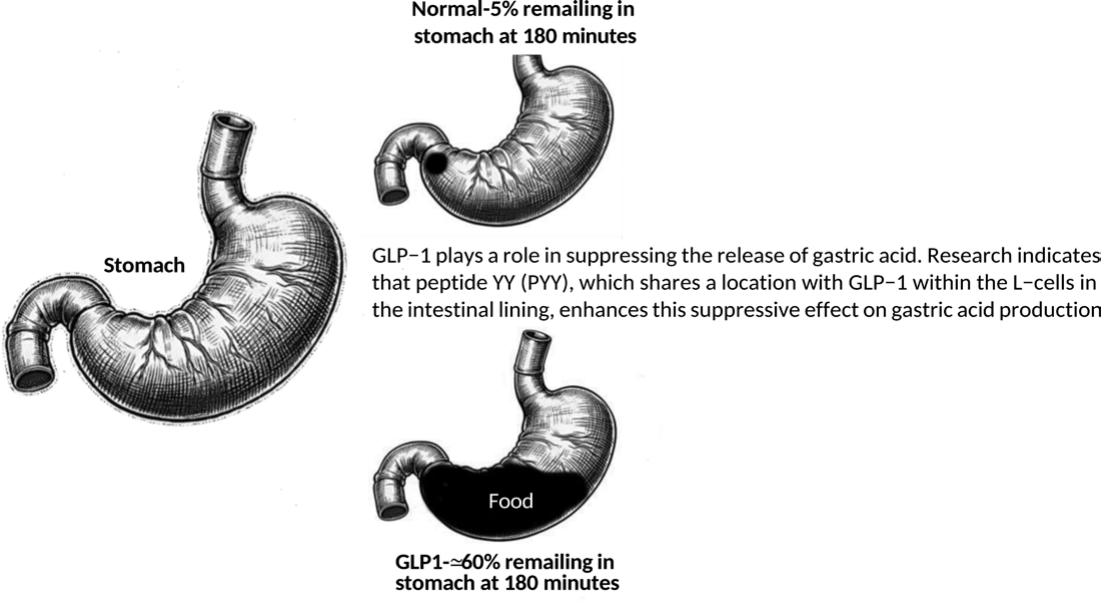
GLP-1 may reduce appetite by enhancing gastric satiety signals, potentially due to a slower rate of gastric emptying. Clinical study by NASLUND and associates using scintigraphy gastric emptying in eight normal male volunteers during intravenous infusion of glucagon-like peptide-1 vs. saline. GLP-1, glucagon-like peptide-1.

The findings above suggest that GLP-1 (Fig. 2), which continues to be explored as a revolutionary treatment for diabetes and obesity, can increase the stomach’s volume both before and after meals and can slow down the emptying of the stomach, without worsening any symptoms after eating in healthy subjects. GLP-1 could dampen vagal cholinergic activity. Further research is warranted to fully understand how GLP-1 causes an increase in the stomach’s volume after meals [6].

## Surgery and anesthesia concerns

### Presurgical context

In the presurgical context, healthcare professionals assess the risk of aspiration by considering a patient’s medical history, including medications like GLP-1 receptor agonists. Precautions, such as fasting before surgery and administering medications to reduce stomach acidity, are taken to reduce the risk of aspiration [7].

It is crucial for people with gastroparesis or those considering treatment options to consult with a healthcare professional for personalized advice based on their specific medical history and circumstances. While ongoing research explores the therapeutic potential of GLP-1 receptor agonists for gastroparesis, caution is warranted in the perioperative setting.

The GLP-1 hormone has significant implications on gastric motility resulting in increased importance for understanding the risk associated with GLP-1 pathway medications during anesthesia Semaglutide, with a half-life of approximately 7 days, requires around 23 days (3.3 half-lives) for its levels to drop to less than 10% of the initial blood level.

However, the impact of withholding the drug for this duration preoperatively on achieving full recovery of gastric motility remains uncertain [8].

### Glucagon-like peptide-1 + anesthesia

While there is limited direct evidence linking GLP-1 to the risk of aspiration during anesthesia, medications on the GLP-1 pathway, like incretin-based therapies, have been associated with gastrointestinal side effects. Patients on GLP-1 receptor agonists may have altered gastrointestinal motility, theoretically increasing the risk of aspiration during anesthesia. The risk is likely multifaceted, influenced by patient-specific factors, the type of anesthesia administered, and the nature of the surgical procedure.

When it comes to the intake of solid foods and nonhuman milk, the current guideline recommendation is to avoid these at least 6 h before elective procedures involving general anesthesia, regional anesthesia, or sedation/analgesia, such as monitored anesthesia care. The Task Force points out that consumption of fried or fatty foods, or meat, could prolong stomach emptying. In such cases, a longer fasting period, perhaps 8 h or more, might be necessary. The specific quantity and type of food eaten should be considered in determining an appropriate fasting duration [9].

A case report highlighted a patient on semaglutide exhibiting significant gastric content despite fasting, indicating the need for caution during anesthesia. A 42-year-old individual diagnosed with Barrett’s esophagus taking a consistent weekly GLP-1 regimenexhibited significant gastric content during a repeat upper gastrointestinal endoscopy and dysplastic mucosa ablation. This occurred 2 months after initiating weekly semaglutide injections for weight loss. Despite an 18-h fasting period, unlike previous procedures, suctioning was necessary before endotracheal intubation [10].

This case highlights the importance of understanding the potential impact of GLP-1 medications on delayed gastric emptying and increased risk of intraoperative pulmonary aspiration of gastric contents.

A potential avenue for addressing this concern lies in the increasing perioperative use of point-of-care ultrasound to assess gastric content. Some previous studies have employed quantitative measures of gastric ultrasounds to identify whether the stomach contains solids, thick liquids, or an excessive volume of clear, which are risk factors for aspiration during anesthesia. Studies have shown that routine preoperative gastric ultrasound can help tailor perioperative management by identifying patients at high or low risk of aspiration, thereby potentially improving patient safety [11,12].

Until additional data is available, a cautious approach is advisable. Patients utilizing GLP-1 for weight loss should be treated as individuals with a full stomach during procedures to mitigate the risk of complications. In the surgical context, patients on GLP-1 medications may require special considerations to mitigate aspiration risk. This includes assessing medical history and taking precautions like fasting and acid-reducing medications.

### Implications in cardiac procedures and surgeries

On the other hand, patients undergoing cardiac surgery are often at high risk for post-op hyperglycemia. A randomized trial compared effects on post-op hyperglycemia for patients receiving 0.5 mg subcutaneous liraglutide on the evening before surgery and 1.2 mg after induction of anesthesia or placebo. Patients who received pre-op liraglutide had significantly lower needs for insulin injections in the post-op settings, additionally, there was no difference in the incidence of postoperative complications, nausea and vomiting or incidence of hypoglycemia [13].

Similarly, another study explored the impacts of using GLP-1 medications in the pre-op setting before coronary artery bypass grafting compared clinical outcomes for patients who received continuous infusion of GLP-1 12 h before coronary artery bypass grafting and continuing for 48 h after the surgical procedure. Results of this study found that patients in the control group without GLP-1 pretreatment required greater incidence of arrythmias and more frequent use of inotropic and vasoactive infusions during the 48 h after the surgery [14].

### Moderate sedation procedures

Recent research suggests that prolonged fasting before certain medical procedures using moderate sedation might not be necessary. Patients scheduled for coronary artery catheterization are usually instructed to fast from midnight before their procedure, leading to a fasting period that lasts at least 6 h and can be longer depending on when the procedure is scheduled. This mandatory fasting can lead to negative effects like discomfort, irritability, dehydration, and a drop in blood sugar levels. However, there seems to be a lack of substantial evidence to support such fasting requirements, especially for patients with low to medium risk undergoing cardiac catheterization.

Regarding risks associated with procedures like emergency coronary artery bypass grafts, the probability of complications such as gastric content regurgitation, aspiration pneumonia, and the necessity for emergency endotracheal intubation is quite low, varying from less than 0.1 to 0.4%. The risk of cardiac arrest is also below 1%. Studies show minimal differences in gastric complications whether patients fast for just 2 h or from midnight before their intervention.

Warner *et al*. [15] noted that the incidence of pulmonary aspiration is very rare, with rates of 0.02% for elective and 0.01% for emergency procedures. However, while these studies comment on moderate sedation procedures in the general population, currently limited understanding exists regarding differences that may be seen in patients on GLP-1 medications.

## Implications for reduction of gastric motility on other medications

Slowing down the movement of the stomach (gastric motility) can have implications for the absorption and onset of action of certain drugs, potentially influencing their effectiveness and safety. With the increasing use of GLP-1 medications for the treatment of type 2 diabetes and obesity, understanding regarding their impact on other medications that require gastric absorption is limited. Below are examples of drug classes and specific drugs that may be impacted by changes in gastric motility, though there are many more [16]:

Several cardiac medications such as platelet inhibitors have been shown to be impacted by decreased gastric motility.

Studies have examined the impact that concomitant use of opioids can influence the bioavailability of oral platelet inhibitors such as clopidogrel and ticagrelor. In contrast, medications such as cangrelor, a parenteral antiplatelet agent, warfarin, and some cardiac medications, such as statins and angiotensin-converting enzyme inhibitors appear to not be as impacted by decreased gastric motility [17,18].

**Fig. 3:**
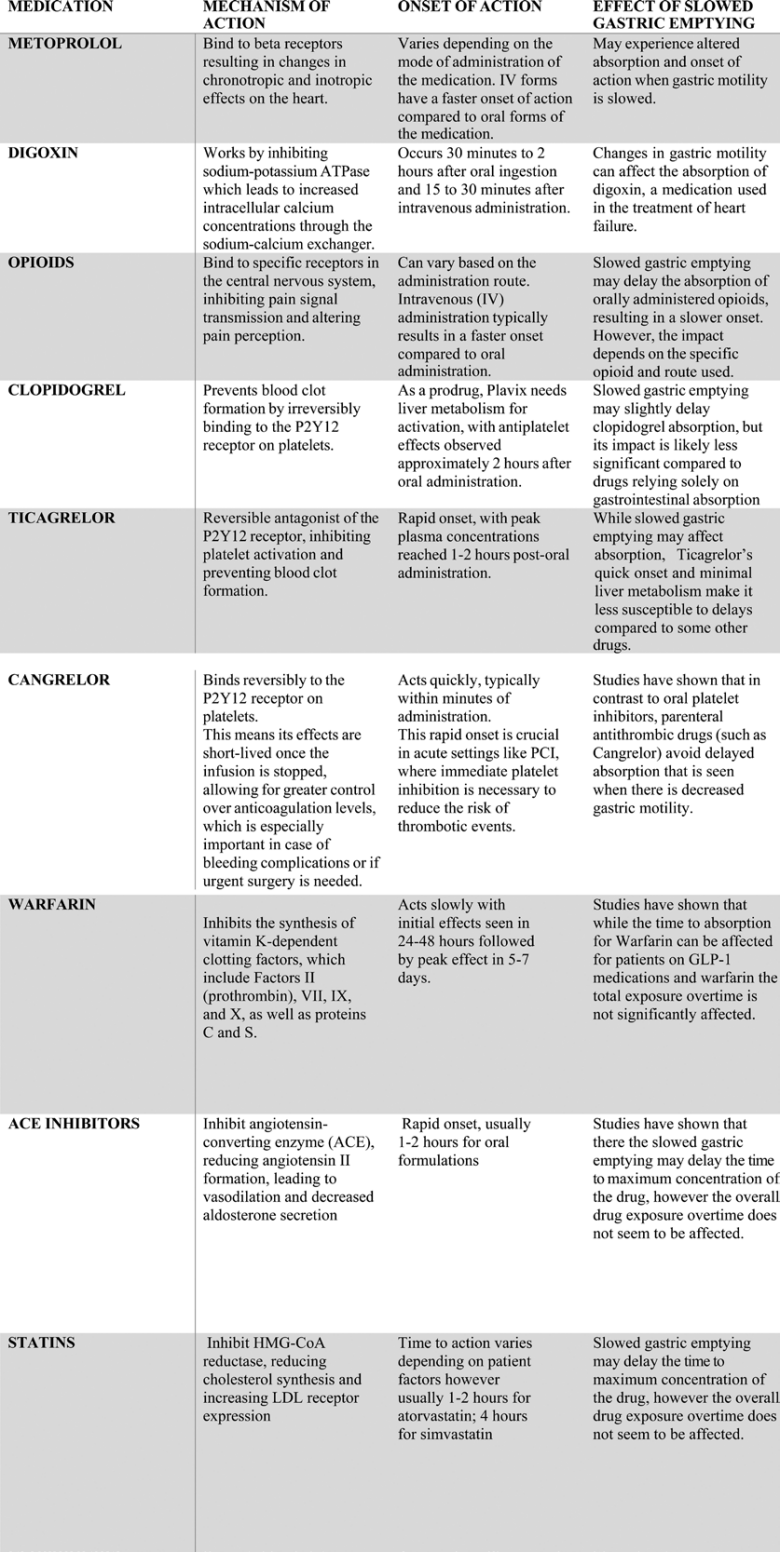
Highlights the mechanism of action, onset of action, and effect of slowed gastric emptying of some commonly used medications in cardiac patients. PCI, percutaneous coronary intervention.

The drugs highlighted above in Fig. 3 encompass a small portion of medications that patients being evaluated for preoperative risk may be taking. While studies have looked at the effects of GLP-1 receptor agonists (RAs) on the pharmacokinetics of these medications the data is still limited. Consideration of individual patient factors, overall health, liver function, and specific medication formulations/routes is crucial. Currently, the American Society of Anesthesiologists recommends holding GLP-1 RAs on the day of the procedure to mitigate risks associated with delayed gastric emptying, such as aspiration [9]. Healthcare providers may adjust treatment plans or opt for alternative administration routes if concerns arise about delayed onset due to gastric issues.

In summary, understanding the interplay between gastric motility and drug absorption is crucial for optimizing therapeutic outcomes. Patient-specific considerations and professional guidance are essential for managing potential delays in drug onset associated with gastric issues. Further studies are needed to better understand the implications of using these medications with GLP-1 receptor agonists.

## Potential therapeutic role for treatment of gastroparesis

Owing to its effects on gastric motility and appetite regulation, researchers have explored the potential therapeutic use of GLP-1 receptor agonists in the context of gastroparesis. However, the evidence is not yet conclusive, and more research is needed to establish the safety and efficacy of these agents for gastroparesis.

The use of GLP-1 receptor agonists in the management of gastroparesis is still an area of ongoing research. Clinical trials and studies are essential to determine the optimal dose, duration, and safety of these medications for people with gastroparesis.

### Conclusion

This paper outlines the complex relationship between GLP-1 and delayed gastric emptying, particularly gastroparesis, and its implications for pulmonary aspiration during anesthesia for various cardiac procedures and surgeries. GLP-1, crucial for glucose homeostasis and satiety, may also affect gastric motility. This is significant for patients undergoing anesthesia for procedures, where aspiration risks are heightened by factors like a full stomach and reduced consciousness. Furthermore, slowed gastric motility also impacts drug absorption, potentially affecting medications such as beta-blockers, digoxin, opioids, and antiplatelet drugs. Unknown bioavailability of these medications may alter the risks associated with surgery for patients. Understanding this interplay is vital for optimal therapeutic outcomes.

In summary, while GLP-1’s role in gastric motility offers potential therapeutic benefits, particularly in diabetes management, its implications for anesthesia and drug absorption necessitate careful consideration and highlight the need for ongoing research. It is unclear currently whether stopping GLP-1 2 weeks before and allowing the drug level to return to normal will help before pending elective surgery or if there are other benefits for patients continuing GLP-1 medications before elective procedures and surgeries.

## Acknowledgements

### Conflicts of interest

There are no conflicts of interest.
